# Low serum 25-hydroxyvitamin D level and risk of urinary tract infection in infants

**DOI:** 10.1097/MD.0000000000004137

**Published:** 2016-07-08

**Authors:** Jianhuan Yang, Guangdao Chen, Dexuan Wang, Minguang Chen, Chao Xing, Bin Wang

**Affiliations:** a Department of Pediatric, Zhujiang Hospital of Southern Medical University, Guangzhou; b Department of Pediatric, The Second Affiliated Hospital & Yuying Children's Hospital of Wenzhou Medical University, Wenzhou, Zhejiang; c Department of Pediatric, Central Hospital of Panyu District, Guangzhou; d Department of Clinical Laboratory, The Second Affiliated Hospital & Yuying Children's Hospital of Wenzhou Medical University, Wenzhou, Zhejiang, China.

**Keywords:** 25-hydroxyvitamin D, infant, urinary tract infection, vitamin D

## Abstract

The aim of the study is to determine whether serum 25-hydroxyvitamin D (25(OH)D) deficiency in infants increased odds of urinary tract infection (UTI). A total of 238 infants including 132 patients experiencing a first episode of UTI and 106 controls, aged from 1 to 12 months, were enrolled. Serum 25(OH)D levels were tested through blood sampling. The serum 25(OH)D levels were significantly lower in cases with UTI than controls. The mean serum 25(OH)D levels were 29.09 ± 9.56 ng/mL in UTIs and 38.59 ± 12.41 ng/mL in controls (*P* < 0.001). Infants with acute pyelonephritis (APN) had lower serum 25(OH)D than those with lower UTI. The multivariate logistic regression analyses showed that serum 25(OH)D < 20 ng/mL (OR 5.619, 95% CI 1.469–21.484, *P* = 0.012) was positively related to an increased odds of UTI. Vitamin D supplementation (OR 0.298, 95% CI 0.150–0.591; *P* = 0.001) was associated with a decreased likelihood of UTI. Vitamin D deficiency in infants was associated with an increased odds of UTI. Interventional studies evaluating the role of vitamin D supplementation to reduce the burden of UTI are warranted.

## 1 Introduction

Urinary tract infection (UTI) is one of the most common infectious diseases in febrile infants during the first year of life,^[1]^ with boys mainly during the first 6 months and girls thereafter^[2]^. *Escherichia coli* is the predominant pathogen found in 90% of girls and 80% of boys at the primary UTI. In recurrences, the proportion of non-*E coli*, for example, *Klebsiella*, *Enterobacteriaceae,* and *Proteus*, is getting higher.^[3]^ Approximately 20% to 30% vesicoureteral reflux (VUR) is found in the children's first febrile UTI, half of these with dilating VUR (grades III–V).^[4]^ There is a strong correlation between febrile UTI recurrence and renal scarring,^[5]^ which may result in progressive renal damage, hypertension, and end-stage renal failure.

Vitamin D has known effects on the immune system. Antimicrobial peptides induced by vitamin D may assist preventing infections.^[6,7]^ Vitamin D may modulate the production of cytokines and suppress inflammation,^[8]^ and thus, reduce the severity of infection. Vitamin D deficiency has been reported in children with sepsis, community-acquired pneumonia, and influenza.^[9–11]^ Van der Starre et al^[12]^ found a correlation between vitamin D deficiency and UTI in adults. However, we are unaware of the role of vitamin D deficiency in infants (first year of life) with UTI. This study was undertaken to determine if there was any correlation between serum 25(OH)D levels and UTI in infants.

## 2 Subjects and methods

### 2.1 Participants

A hospital-based case-control study was conducted in the Urology Department of Pediatrics at Wenzhou Medical University Hospital, China, between August 2014 and July 2015. In total 132 patients experiencing a first episode of UTI, aged from 1 month to 12 months, were enrolled. Inclusion criteria were as follows: (a) first episode of UTI, (b) presence of clinical symptoms and signs such as fever, poor feeding, vomiting, foul-smelling or cloudy urine (c) pyuria, (d) positive urine culture. Exclusion criteria were as follows: (a) neonate younger than 1 month of age; (b) infants who were identified congenital anomalies of the kidney and urinary tract (CAKUT) were excluded; (c) urinary stones; (d) chronic renal failure. Controls were 106 cases of healthy infants, living in the same area and attending the clinic examination during the study period.

This study was approved by the ethics committee of Wenzhou Medical University, and informed consent was obtained from all parents before study entry.

### 2.2 Demographic characteristics

A structured questionnaire was used to obtain information concerning age of the infant, gender, height, weight, feeding history, exposure of sunlight, supplementation of Vitamin D. Feeding types included breast milk, formula, or mixed feeding (both breast milk and formula). Sun exposure behavior was assessed by recording the duration of direct sun exposure in a week (h/wk). Information of vitamin D supplementation was recorded including the dose and duration of vitamin D intake.

Infants who had a minimum daily intake of 400 IU (international unit) of vitamin D for >1 month were divided into “vitamin D supplementation group.”

### 2.3 Urine samples

Urine samples were obtained by the midstream clean catch or by suprapubic catheterization. Pyuria was defined as ≥ 5WBCs (white blood cell) per high-power field. Positive urine culture was defined as >10^5^ CFU/mL (colony forming unit) of a single pathogen in a midstream urine sample, or 10^4^ CFU/mL via urinary catheterization.

### 2.4 Blood samples

Venous blood specimens were collected from both cases and controls. Serum 25(OH)D levels were determined in both UTIs and controls, whereas serum C-reactive protein (CRP) and complete blood count determined only in UTIs. A commercial radioimmunoassay kit (Roche Diagnostics GmbH, Vitamin D total) was used to measure serum 25(OH)D levels. Levels of 25(OH)D were categorized as sufficiency (≥30 ng/mL), insufficiency( < 30 ng/mL but≥20 ng/mL), and deficiency (<20 ng/mL).^[13,14]^ Hypovitaminosis D was defined as insufficiency or deficiency of Vitamin D.

### 2.5 Imaging examinations

Renal ultrasonography was performed in all patients within the first 2 days of admission and ^99m^Tc -dimercaptosuccinic acid (DMSA) scan within 5 days of admission. An abnormal DMSA scan suggesting APN was defined as focal or diffuse areas of decreased uptake.^[15]^ Based on the result of DMSA renal scan, patients were divided into APN and lower UTI groups. Voiding cystourethrography (VCUG) was performed 1 to 2 weeks after diagnosis of APN by DMSA renal scan and control of the acute infection to verify the presence of vesicoureteral reflux (VUR). The VUR was graded I–V according to the International Reflux Study in children.^[16]^

### 2.6 Statistics

Data was analyzed using SPSS (Version 19.0; SPSS Inc, Chicago). The normality of continuous data was assessed by the Kolmogorov–Smirnov test. Normal distribution variables were evaluated by *t* test. Skewed distribution variables were assessed by Mann–Whitney *U* test. Categorical variables were compared by the chi-square test. Patient characteristics associated with UTI in univariate analysis (*P* < 0.05) were included in the multivariable model. We used multivariate logistic regression analysis to assess the influence of these risk factors on UTI. Results were expressed as mean ± standard deviation (SD), odds ratio (OR), 95% confidence interval (CI), and *P* value. *P* < 0.05 was considered statistically significant.

## 3 Results

### 3.1 The clinical characteristics of the participants

In total, 132 (70 boys, 62 girls) patients and 106 (56 boys, 50 girls) controls were enrolled. The mean age of patients and controls was 7.29 (SD 3.06) months and 7.09 ( SD 3.25) months, respectively. There was no significant difference between 2 groups in age and gender (*P* > 0.05, Table 1). In total 53.79% of patients and 59.43% of controls received exclusive breastfeeding, and there was no statistically difference between 2 groups (*P* = 0.383, Table 1). Only 56.06% of patients had vitamin D supplementation (400 IU/day) for >1month, and the percentage of vitamin D supplementation in patients was significantly lower (*P* = 0.005, Table 1) than in controls (73.58%). The duration of sun exposure was shorter (*P* < 0.001, Table 1) in patients (mean 2.57 h/wk, SD 0.87 h/wk) than in controls (mean 3.08 h/wk, SD 1.04 h/wk).

**Table 1:**
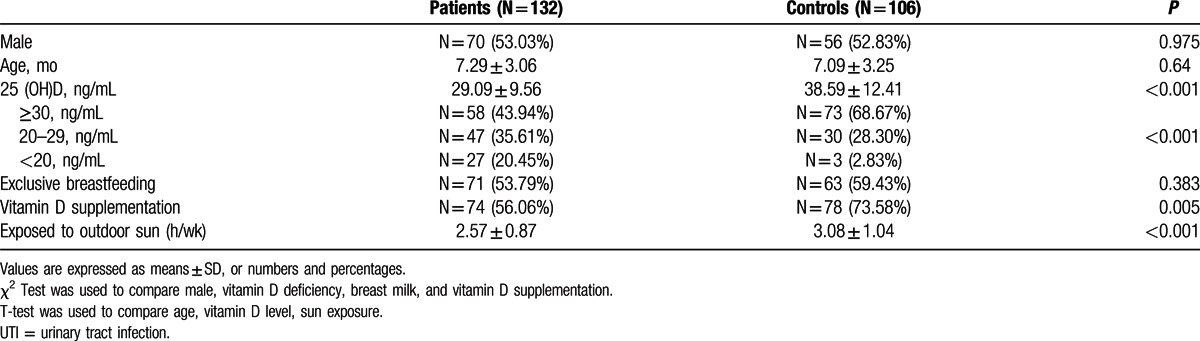
Basic characteristics of infants with and without UTI.

The mean serum 25(OH)D levels were 29.09 ± 9.56 ng/mL in patients and 38.59 ± 12.41 ng/mL in controls. The serum 25(OH)D levels were significantly lower in UTIs than controls (*P* < 0.001, Table 1). 35.61% of UTIs were 25(OH)D insufficiency, and 20.45% were 25(OH)D deficiency. Although 28.30% of controls were 25(OH)D insufficiency, only 2.38% were 25(OH)D deficiency. The rate of hypovitaminosis D (both insufficiency and deficiency) was significantly higher in UTIs than controls (*P* < 0.001, Table 1).

### 3.2 Vitamin D status in different types of UTI

Infants with APN had lower serum 25(OH)D than those with lower UTI (Table 2. *P* = 0.015). The mean serum 25(OH)D levels were 27.69 ± 9.29 ng/mL in the APN group and 31.98 ± 9.59 ng/mL in the lower UTI group. Meanwhile, serum CRP and WBC counts were higher in the APN group than the lower UTI group (*P* < 0.05). There was no statistically significant difference in serum 25(OH)D, serum CRP, and WBC counts between the APN group with VUR and the APN group without VUR (Table 3*P* > 0.05).

**Table 2:**
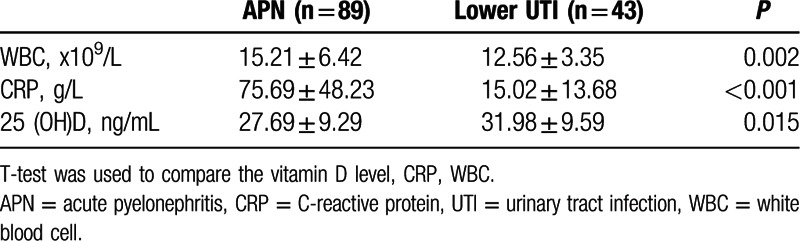
Comparison of laboratory findings between the APN group and the lower UTI group.

**Table 3:**
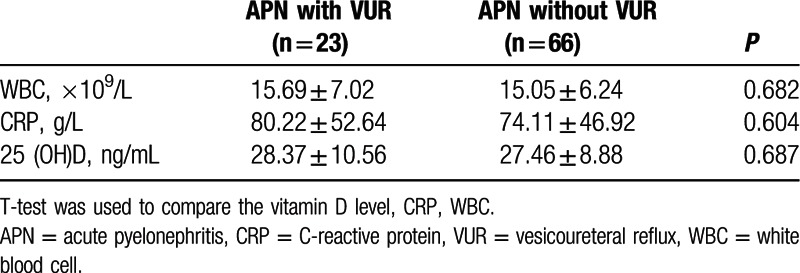
Comparison of laboratory findings between the APN with VUR group and the APN without VUR group.

### 3.3 Factors associated with UTI in multivariate analysis

Variables showing statistically significant association with UTI in univariate analyses were set into a multiple logistic regression analyses. Formula was included as it may affect the serum 25(OH)D level, although it was not independently associated with UTI. The 20 ng/mL cutoff was used for the serum 25(OH)D level because few control infants (only 2.38%) had vitamin D deficiency. It was reported that^[17]^ the average duration of sun exposure for infants in Shanghai, China, was 3 h/wk. So the cutoff of sun exposure was 3 h/wk in our study. The multivariate logistic regression analyses showed that serum 25(OH)D <20 ng/mL (Table 4 odds ratio [OR] 5.619, 95% confidence interval [CI] 1.469–21.484; *P* = 0.012) was positively related to an increased odds of UTI. Vitamin D supplementation (OR 0.298, 95% CI 0.150–0.591; *P* = 0.001) was associated with a lower odds of UTI.

**Table 4:**
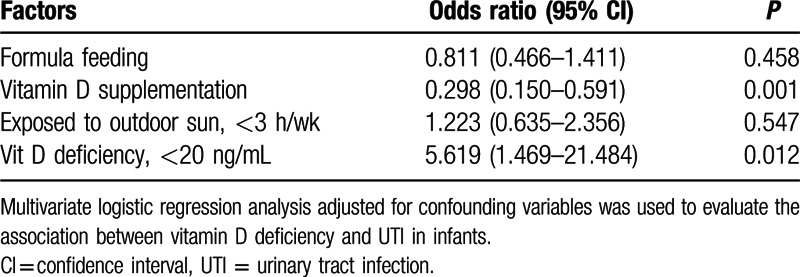
Factors associated with UTI in multivariate analysis.

## 4 Discussion

We identified a statistical prevalence of vitamin D deficiency in infants with urinary tract infection in this study. Previous studies have shown that vitamin D has important roles for both innate^[18]^ and adaptive immune responses.^[19]^ Vitamin D has been linked to innate immune responses mainly by increasing the neutrophilic motility and phagocytic function.^[20]^ In addition, antimicrobial peptides induced by vitamin D may defend against bacterial infection.^[21]^ Low vitamin D is the consequence of a chronic inflammatory process caused by persistent infection.^[22]^ Excess 1,25(OH)2D is produced in an effort to upregulate the VDR to transcribe AMPs, and 25(OH)D is rapidly metabolized in the process, resulting in a low serum level.^[23]^ It is asserted that low levels of 25(OH)D accurately reflect vitamin D status.^[24]^ Lots of studies have verified the relationship between vitamin D deficiency and respiratory tract infections,^[20,25–27]^ but few study demonstrated the association between vitamin D deficiency and UTI in infants. Nseir et al^[28]^ found that recurrent UTIs in premenopausal women are associated with vitamin D deficiency. More recently, Tekin^[29]^ found that vitamin D deficiency may be a risk factor for UTI in children. Our study extended these results, suggesting that vitamin D deficiency was an increased odds of UTI in infants, and vitamin D supplementation was a lower odds of UTI. If confirmed with intervention trials, our findings may have important public health implications.

We identified that the serum 25(OH)D level in infants with UTI was significantly lower than that in healthy infants (*P* < 0.001), and the rate of vitamin D deficiency was significantly higher in infants with UTI(*P* < 0.001). Similar results were found in the recent study,^[29]^ but the serum 25(OH)D level was much lower in children with UTI(11.7 ± 3.3 ng/mL in previous study vs 29.09 ± 9.56 ng/mL in our study). The difference may result from different population (race, gender, age), study design, serum 25(OH)D measurements. We also found that infants with APN had lower serum 25(OH)D level than infants with lower UTI(*P* = 0.015). Based on the result of VCUG, patients with APN were divided into 2 groups (VUR group and non-VUR group). We found that the serum 25(OH)D levels in both groups were not statistical difference (*P* > 0.05). But we failed to show an association between the serum 25(OH)D level and classification of VUR because of small sample size in the VUR group.

However, recent study shows that serum 25-(OH)D is an unreliable biomarker of vitamin D status after acute inflammatory insult.^[30]^ Hypovitaminosis D may be the consequence rather than cause of chronic inflammatory diseases.^[30]^ Our study revealed a correlation between vitamin D deficiency and UTI in infants, but the causation was uncertain. We presume that vitamin D deficiency may worsen existing immune and metabolic dysfunctions in infants, leading to worse outcomes.

In the multivariate analysis, vitamin D supplementation decreased the likelihood of UTI. As expected, most infants who were exclusive breastfeeding in China had low serum vitamin D levels. Recent studies indicate that the prevalence of serum 25(OH)D <30 nmol/L is high and worldwide in breastfeeding infants, and lack of sun exposure and vitamin D supplementation have been considered as contributing factors.^[31,32]^ Breast milk may not provide enough vitamin D for infants, especially when the mothers are also vitamin D-deficient.^[33,34]^ Due to insufficient exposure to sunlight and a diet not enriched with vitamin D, pregnant women suffer from vitamin D deficiency and lead often to birth of neonates with the same deficiency.^[35]^ Therefore, additional vitamin D is needed from sunlight or vitamin D supplementation for both mothers and infants. It is now recommended that all infants have a minimum daily intake of 400 IU of vitamin D beginning soon after birth.^[36,37]^ The most recent Institute for Organization Management (IOM) report recommends 400 IU/day for infants <1 year and 600 IU/day for children aged 1 to 8 years.^[38]^ Though sun exposure was not a protective factor associated with UTI in multivariate analysis, it may correlate with vitamin D status in infants. It is in view of the recommendation to restrict exposure of infants aged <6 months to direct sunlight.^[39]^ We found that the duration of sun exposure was shorter in UTIs(2.57 ± 0.87 h/wk) than controls (3.08 ± 1.04 h/wk) in univariate analyses, but the association between sun exposure and UTI needed a further study.

This study was conducted in infants in our city and may not be generalizable to other populations. Studies evaluating other pediatric populations are warranted. We identified that infants with vitamin D deficiency were at an increased odds for UTI, whereas vitamin D supplementation was associated with a lower odds of UTI. This study provides evidence in support of future interventional trials to determine if vitamin D supplementation decreased the development of UTI.

## Acknowledgments

The authors thank all the parents and infants who participated in the study, and the Yuying Children's Hospital of Wenzhou Medical University.
